# Exploring ‘quality’ in science communication online: Expert thoughts on how to assess and promote science communication quality in digital media contexts

**DOI:** 10.1177/09636625221148054

**Published:** 2023-01-31

**Authors:** Birte Fähnrich, Emma Weitkamp, J. Frank Kupper

**Affiliations:** Freie Universität Berlin, Germany; University of the West of England, UK; Vrije Universiteit Amsterdam, The Netherlands

**Keywords:** digital media, quality, science communication

## Abstract

In recent years, the public visibility of science has greatly increased. In the digital media landscape, a wide range of players is now engaged in science communication via various online channels. While these developments offer opportunities, they also entail risks for the quality of science communication. This study explores how the quality of science communication can be assessed and promoted in the increasingly complex digital ecosystem. A two-wave survey with international science communication experts served as a basis to develop a quality framework for digital science communication and to formulate strategies to promote the quality of science communication online. Besides these outcomes, results hint at blind spots in the discourse of science communication quality that demand further investigation and reflection.

## 1. Introduction

Today, science-related content is communicated, perceived and interpreted largely online (Newman et al., 2020). We encounter science on diverse platforms, such as a daily newspaper’s newsfeed, Facebook or Instagram posts from NGOs or other activists, scientists’ podcasts on Spotify or video clips on TikTok, the posts of science enthusiasts on Reddit, science sceptics’ videos on YouTube, or tweets from lobby groups and social movements containing scientific charts and figures on Twitter. This patchwork of science communication online, which we define broadly as ‘all forms of communication about science-related topics via digital media’ (Fähnrich, 2021), raises questions about how science content is searched for, found, consumed, perceived and understood, aspects that are dependent on the communication context (Kahan et al., 2017).

Global challenges, such as the COVID-19 pandemic, climate change or security issues, have pointed to the societal relevance of science communication which has been considered as an important prerequisite for responsible decision-making (Von Winterfeldt, 2013). Online communication has been said to lower the hurdles for scientists to undertake public engagement (Jünger and Fähnrich, 2020), though open science or publishing models may not truly increase accessibility. In contrast, recent debates around fake news, misinformation, science denial or the so-called COVID-19 ‘infodemic’ point to the threats and challenges that the digital media environment pose for public communication in general (van Bavel et al., 2020) and science communication in particular (Zarocostas, 2020), which also affect the quality of science communication. Whereas in traditional media, editorial standards and regulation are typically applied (Weitkamp et al., 2021), this is not necessarily the case for emerging communicators who, however, also contribute to the public perception of science. From an audience perspective, studies from Germany, to name but one example, indicate users fail to recognize journalistic content on the web (Neuberger, 2014) or do not care about the sources of the news that they consume (Hölig and Hasebrink, 2013). This lack of critical engagement on the part of users highlights the need for quality assessment criteria which can be adopted by content creators.

We argue that the maintenance of quality has become of central concern and reflecting upon the quality of science communication is of vital importance. To this end, the study presented here investigated quality indicators that could be conceptualized in the digital science communication ecosystem.

This was addressed through the following sub-questions:

1. What criteria can be applied to assess the quality of science communication online?
2. How do these indicators differ from traditional evaluation criteria or across platforms?
3. How can quality standards for science communication be promoted in an increasingly complex digital media environment?

To respond to these questions, we present data from an exploratory study^1^ surveying international science communication scholars. Carried out in two waves, the study adopted a Delphi design and included 31 scholars who shared their perspectives on science communication quality through two consecutive online surveys. On the one hand, the results of the study shed light on the dimensions in which quality of science communication online can be conceptualized, and accordingly, how quality could be assessed and promoted. On the other hand, they hint at blind spots in the academic discourse of science communication and science communication quality that demand further reflection.

## 2. Science communication quality in digital contexts

Digital media are changing the role of journalism which has lost its position as the primary information intermediary to become one source of scientific information among many (Bauer, 2013; Newman et al., 2020). These changes have opened new spaces for science communication which have in turn enabled new voices to contribute to the science communication landscape (Pearce et al., 2019; Trench, 2007; Weitkamp et al., 2021). This has led to a blurring of roles between consumers and producers of information (Bruns, 2008); not only can traditional knowledge holders, such as research institutes, governments and museums, now reach the public directly (Koivumäki and Wilkinson, 2020) but so can individuals we might consider ‘non-professionals’ in a science communication context, allowing audiences to become active participants (Schäfer, 2017). Digital tools foster collaboration and interaction that enables and simplifies ‘reciprocal, multi-level and sequential communication’ (Neuberger, 2014: 567). These affordances of digital technologies mean that a wider range of individual and institutional actors can become publicly visible in online channels and are able to affect the public discourse and opinion (Kaiser et al., 2017). These changes present challenges when it comes to assessing quality in science communication.

Enabling a greater diversity of voices to enter the public discourse, digital media have also changed the way information is produced and consumed. Now, ‘news articles rival with user-generated content like blog posts, personal status updates, song recommendations, or cat pictures for the user’s attention’ (Kaiser et al., 2017: 10). News articles become part of a patchwork of content whose sources are (at least partly) unrecognizable and whose credibility is often difficult to assess (Neuberger, 2014). Within the science communication landscape, a huge variety of societal actors, such as universities, activist groups, corporations, political actors, bloggers and science sceptics, are using digital media, communicating about science in the online public sphere (Allgaier, 2019; Fähnrich, 2021; Metag and Schäfer, 2019). Such developments could be seen to foster democratization of science (Kahan et al., 2017), though they come with risks, particularly in relation to quality standards (Peters et al., 2014). Furthermore, the speed with which information can be shared online encourages competition, potentially at the expense of accuracy. At the same time, the archival nature of the Internet can mean that new information sits alongside obsolete information, a problem identified during the COVID-19 pandemic (Lu et al., 2021).

These changes in media and public communication have led to academic and political scrutiny of the quality of science communication (e.g. Nisbet and Scheufele, 2009). Drawing on the assumption that scientifically informed knowledge is an important prerequisite for responsible decision-making, it is regarded as desirable that citizens use high-quality journalistic or media content to be adequately and accurately informed about relevant topics (Dohle, 2017). Thus, understanding how to ensure the quality of science communication becomes an important facet of any drive to improve the quality of interactions between science, media and society.

Defining quality in science communication is challenging (Bucchi and Trench, 2014) and typically draws on related fields, such as (science) journalism (Gibson et al., 2015; Lacy and Rosenstiel, 2015; Meier, 2019), including medical reporting (Zeraatkar et al., 2017) and environmental journalism (Rögener and Wormer, 2017). In previous research, different models and frameworks have been developed to tackle the vagueness of the concept but less so in the context of digital science communication. Scholars have pointed to a huge variety of definitions, the relativity and dynamics of the concept, and related difficulties to assess and evaluate communication quality (Lacy and Rosenstiel, 2015; Neuberger, 2014; Rögener and Wormer, 2017). There is agreement that quality cannot be defined ‘objectively’ but that any quality assessment mirrors the expectations of certain actors (producers and users) towards certain media content. Previous research has examined the quality of public communication from different sides: In a demand perspective, the focus is on the interaction between the needs and requirements of media users and the respective media content (Dohle, 2017; Urban and Schweiger, 2014). From a production perspective, those who produce media content specify and apply characteristics that are associated with high or low quality (Gertler, 2013). In both, quality is a ‘matter of degree. It is not as simple as having or not having quality’ (Lacy and Rosenstiel, 2015).

In a different reading, quality is associated with certain normative requirements and standards that communication should meet. Given the context dependency of quality assessment, the question who should develop these standards is vital. Public communication quality has typically been examined from the perspective of experts (e.g. scientists) or the producers of content (e.g. journalists), though a few studies have explored audience assessments of quality (Dohle, 2017). Quality criteria have also been explored for the related field of public relations (Pieczka, 2002). In broader digital contexts, studies are emerging that explore quality criteria (Chai et al., 2009), including automated approaches (Shah et al., 2009). Other studies investigate the criteria readers use for their quality assessment of websites (Eysenbach and Köhler, 2002) and Facebook (Lee et al., 2018). To our knowledge, only one study has explored quality in the context of science communication in general, focusing on accuracy, style and engagement (Olesk et al., 2021), but fails to consider how such indicators could be implemented or what the implications of such indicators should be for wider science communication discourses.

In a digital context, with ‘content that has been created by users from different backgrounds, for different domains and consumed by users with different requirements’ (Chai et al., 2009: 791), defining and assessing communication quality is even more complex and challenging. We anticipate that criteria for judging science communication quality will depend on the platform (e.g. Facebook, legacy media) and communicator (e.g. blogger, scientist), and may be assessed by different actors (e.g. readers, scholars) differently. Thus, assessing the quality of scientific content becomes complex and challenging. However, as COVID-19 has shown, this challenge needs to be addressed urgently to enable science communication to enhance public discourse on science and technology issues.

## 3. Method

### Approach

In previous research exploring quality, expert panels have played an important role (Gertler, 2013). Following this tradition, we surveyed a panel of science communication experts. The exploratory study aimed at generating ‘reasonably reliable statements for questions [of science communication quality] about which only incomplete knowledge, unsubstantiated hypotheses or mere assumptions exist’ (Steinmüller, 2019: 34). The research design consisted of two waves of surveys to explore how science communication quality can be assessed and promoted in the digital media landscape. We are thus following established methods used in previous quality research, in which expert panels have played an important role (Gertler, 2013).

The expert panel consisted of international researchers whose work addresses (digital) science communication. Such a panel was considered most appropriate to respond to the questions in focus, as they have an overarching perspective on developments in the digital transformation of science communication. Based on their research experience, science communication scholars can objectively evaluate developments in digital science communication, and related quality issues and demands. Although we recognize the value of practitioner perspectives, to ensure comparability, only researchers were included in the study. We consider that experts are likely to exhibit a level of consistency in their epistemological perspective on the subject matter. Scholars were invited to participate when they had published on issues related to science communication in digital contexts and science communication quality. We searched for respective English language publications using a search string entailing the terms ‘digital’, ‘online’, ‘social media’ and ‘quality’ in connection with science communication, science journalism and public engagement on the Web of Science database. On this basis, 70 scholars were approached and 31 agreed to participate in the two-wave study. Meanwhile, 26 participants completed the questionnaire in Wave 1 and 19 took part in Wave 2. Participating scholars represented 17 different national perspectives comprising Austria, Australia, Brazil, Denmark, Estonia, Germany, Ireland, Israel, Italy, Japan, Netherlands, New Zealand, Norway, South Africa, Switzerland, United Kingdom, and United States. Two-thirds of participants were full or associate professors, one-third were PhD students, post doc researchers or assistant professors. Experts had a background in communication science, science and technology studies (STS), media studies, political science, psychology and related fields. The panel consisted of approximately two-thirds men and one-third women. Data collection for Wave 1 took place between November 2019 and January 2020. Wave 2 was conducted between May 2020 and June 2020.^2^ The surveys were conducted anonymously with the tool SoSci Survey.

### Research design and data collection

Considering the gaps in research on quality in science communication, the study took an exploratory and open approach. We created an anonymous, iterative, interactive and domination-free setting to allow the experts to deal effectively with the issue of science communication quality over a longer period of time (Linstone and Turoff, 1975; Niederberger and Renn, 2019; cf. Steinmüller, 2019). Therefore, the study encompassed two waves, whereby the second wave built on the results of the first.

Questionnaires for both waves encompassed a range of open and (for Wave 2) fewer standardized questions to enable a comprehensive perspective on factors that scholars associate with science communication quality. Experts were asked for their judgement using predictive questions (e.g. to outline prospective developments in digital science communication), normative assessments (e.g. sources from which to derive standards) and instrumental questions (e.g. implementation and evaluation of indicators) (Steinmüller, 2019; Surowiecki, 2004).

To orient participants to the topic, the Wave 1 questionnaire initially asked how they would define science communication online. Next, they were asked for their assessment of the most important quality criteria in online science communication, about the fields of reference that could be used to define science communication quality (e.g. journalism, PR, audience research), and whether quality could be assessed in science communication and how this could be done. The final question explored promotion of quality criteria for science communication (see supplemental materials).

The second questionnaire followed the same structure but sought to summarize, complement, consolidate and reflect the initial findings, by presenting and explaining the results of the first survey. Participants were asked to rank results to evaluate outcomes and to comment on authors’ interpretations. To start with, the second questionnaire proposed a broad definition of science communication in digital contexts which encompasses ‘all forms of communication about science-related topics via digital media’. This conception resulted from Wave 1 (cf. Fähnrich, 2021). Participants were invited to comment on this definition. We then presented a map of quality criteria (cf. section ‘Data analysis’). This allowed participants to reflect on categories derived from Wave 1 and add further categories as needed. Participants were then asked to identify criteria that they felt could be generalized to online science communication and also to consider five different settings presenting science communication in an online context. These included communication not only from science and science journalism but also alternative formats and communicators, such as NGOs or political actors. Experts were asked to select two settings and to discuss the differences these might present in terms of quality indicators. In the final section, we presented options for the promotion of science communication quality drawn from participants responses in Wave 1. We asked participants to explain which approaches were already used and which they considered to be most effective. Respondents were also asked about the role of science communication professionals and scholars in the promotion of science communication quality standards.

### Data analysis

To analyse the data collected in Waves 1 and 2, the situational analysis approach developed by Clarke (2003) was applied. Derived from grounded theory, situational analysis is a method that allows complex social phenomena (‘situations’) to be explored. Clarke et al. (2018) explain situations as ‘a somewhat enduring arrangement of relations among many different kinds and categories of elements. . .’ (p. 17). As an interpretative method, situational analysis contributes to a ‘big picture analysis’ (Clarke et al., 2018: 150). It works with heuristic ‘maps’ which are developed to analyse and interpret the data. Three types of maps (situational maps, social world/arena maps, positional maps) are distinguished that follow different logics and thus help to uncover different aspects in the research material (Clarke et al., 2018).

To address Research Question 1, we used situational maps which display major elements in the situation of inquiry and provoke analysis of relations among them (Clarke et al., 2018: xxiv). Answers of the participants to the open questions about relevant quality criteria differed widely. Some responded with brief lists of criteria, others provided more extensive explanations and gave examples. For the analysis, the answers were grouped question by question and numbered consecutively per participant. The analysis process (Table 1) then followed the protocol developed by Clarke et al. (2018). In the first step, all elements of interest were reduced to the central statements on quality criteria and compiled in an unordered manner in a map (‘messy situational map’; cf. Clarke et al. 2018). In the following steps, the elements were grouped together in a condensed form. Multiple entries of the same or similar categories were combined. This map was further structured into an ordered situational map and headers applied. This ordered situational map (meta-criteria and sub-categories) was also used in the second wave and led to additional criteria and assessments (cf. section ‘Research design and data collection’). Eventually, a condensed map was developed. Although not an essential element of a situational map as outlined by Clarke (2003), lead questions were phrased to summarize the sets of categories and to make results more tangible.

**Table 1.** Data analysis process.

Wave	Element	Analysis step
1	Original quote	Grouping and numbering of responses per question
1	Core quote	Reduction to key message, unordered listing in ‘messy’ situational map
1	Group of elements	Inductive ordering of categories, development of ‘ordered’ map
1/2	Meta-criteria	Generation of ‘headings’ for the groups of categories
2	Revision	Adaption of groups and meta-criteria based on results of Wave 2
2	Lead questions	Translation into questions

To address Question 2, analysis focused on which quality criteria apply to different science communication phenomena to reduce the complexity of quality assessments of science communication in online settings (see section ‘Research design and data collection’). This analysis was carried out with the help of a so-called social world/arena map. These maps are used to display the most important actors (social worlds, organizations, institutions, etc.) and discourse arenas, normative settings or cultural orders in which they are involved. The social world map was thus applied to carve out the differences between situational settings (e.g. quality criteria for a scientist’s podcast vs criteria for a government health campaign).

Finally, a positional map was used to address the third research question: how standards for quality in science communication could be promoted and ensured. A positional map plots answers along two axes (formal–informal, direct–self-regulatory). Here, too, the first wave provided a basic exploration, while the focus of the second wave was on supplementing, evaluating and commenting on the authors’ interpretation.

The maps developed in the context of the situational analysis serve primarily to gain an understanding of the research process and are only used selectively for the presentation of results (cf. Clarke et al., 2018). Throughout, maps generated in Wave 1 were reconsidered and revised, considering the responses to the Wave 2 survey. Results displayed in the article are an integrated interpretation of both waves of the study. Original quotes illustrate our findings.

## 4. Results and discussion

### Online science communication quality framework

To investigate experts’ views on quality indicators, we asked experts for criteria that they would associate with science communication quality in the complex digital media environment. Our open question led to an extensive and diverse list of more than 50 criteria that experts considered relevant. Their responses mirrored their different approaches towards and conceptions of science communication. On the one hand, ‘traditional’ criteria relating to science journalism (e.g. Meier, 2019) or science itself (e.g. accuracy, objectivity or transparency) were emphasized, with respondents arguing that these would matter regardless of the context. Some experts argued that ‘factual and scientific accuracy stand out as some of the most important and most widely applicable criteria’ (W1, P18),^3^ reasoning that ‘with mis/disinformation emerging as problems in science, it seems critically important to place an emphasis on determining the quality of content so as to make sure it is not fabricated or false’ (W1, P11). In contrast, it was acknowledged that the transformation of public communication would require rethinking quality standards:

In the dominant science-centric view of two or three decades ago, accuracy in information was the primary, if not exclusive, criterion. Partly through shifts in thinking about models of science communication, this very restrictive basis of assessing quality lost validity. But there was not much effort given to developing alternative criteria. (W1, P6)

Some responses focused more strongly on the competitiveness of the online environment and claimed interactivity and appeal to be increasingly important (also seen in Eysenbach and Köhler, 2002; Olesk et al., 2021). Factors relating to entertainment were mentioned: ‘it would be useful to include ‘pleasure’ – in line with the entertainment and fun in intention of the actors’ (W2, P19); these aspects have been somewhat overlooked in prior research (Afful-Dadzie et al., 2021). Quality was linked to effectiveness, with quality assessment dependent on objectives and targeted audiences:

Quality criteria depend on the intent of the communication enterprise [. . .] Even something as superficially simple as ‘accuracy’ as a quality criterion might not be important if your goal is to inspire people to act. Being wrong could motivate certain audiences to engage with the material more stridently than being correct. (W1, P1)

Experts suggested linking different quality dimensions: ‘if I was assessing a science communication product I would look first and foremost to assess that it is truthful (accurate, impartial, holistic) and story driven (engaging, values oriented, audience focused)’ (W2, P2). Overall, the results show that experts hold quite different perspectives on quality criteria.

Based on this diversity of responses, we refrained from developing a catalogue of fixed and narrow quality criteria as have been developed, for instance, in the context of science journalism (Rögener and Wormer, 2017) or science museums (Olesk et al., 2021). Instead, and in line with several experts who suggested that ‘quality criteria should be multi-dimensional’ (W1, P22), we propose meta-criteria for quality assessment. Working with such an approach seems appropriate given the diversity of science communication online. An orientation towards meta-criteria also accommodates the fact that science communication works at the intersection of science and society and that actors involved hold a wide variety of norms, motives and orientations (Wilkinson et al., 2021).

We integrated, ordered and grouped quality criteria around five pillars. These present complementary meta-criteria that work as a broad and flexible quality framework that can help to assess communication quality within the diverse, complex and constantly changing science communication ecosystem. The proposed framework (see Table 2) contains the following meta-criteria: content, presentation, technology, context and procedure. *Content criteria* refer to characteristics of the information per se. These criteria encompass aspects, such as accuracy, objectivity, completeness and truthfulness, which are also found in (science) journalism and scholarly communication. Relevance was added as a new content criterion, although its assessment was considered to depend on context. *Presentation criteria* refer to the approach used to communicate science content and include language characteristics, such as readability and comprehensibility, in the presentation of multiple perspectives, but also aspects of framing (‘Need to respond to schemas that audiences care about’ (W2, P15)). Additional criteria include reading appeal and whether science communication is engaging, including visuals (W2, P8). Although presentation criteria have been discussed in the context of science journalism, their importance is rising with the increasing competition for public attention in the digital media environment: ‘If audiences don’t pay attention to something, it kind of doesn’t exist’ (W1, P11). This issue was also identified in other contexts (Olesk et al., 2021). These questions of presentation are closely connected to *technical criteria* which are considered to strongly affect quality assessments. In this category, the adoption of specific platform criteria (e.g. regarding different standards on social media platforms, such as Facebook or Reddit) and opportunities for interactivity are associated with quality: ‘there should be opportunities for dialogue/feedback and/or participation by the audiences’ (W1, P10). Moreover, overall characteristics of online communication which become apparent in the level of hybridity and media convergence, for example, through links, are considered important. *Context criteria* deal with the institutional and moral framework of science communication online. As one respondent put it: ‘an important caveat is that a variety of ethical, social, legal concerns are extremely relevant to healthy science communication, yet operate somewhat independently of objective scientific facts’ (W1, P18). Context criteria not only encompass facets, such as the underlying purpose or motivation for the communication, but also the reliability of evidence. Aspects such as legitimacy, and expertise and reputation of sources fall into this category, ‘with a focus on “who” communicates . . . “credibility” (encompassing perceived expertise and trust)’ (W2, P7) is considered important. Participants added that context included the political and social background and how long the information might be relevant. From a user perspective, assessing context criteria is especially challenging as they demand clarity and transparency (of authors, sources, backgrounds) of communication. *Procedural criteria* follow a slightly different logic and refer to the aspects of planning and producing (strategic) science communication. ‘If the goal is to build public support for science, we should not be afraid to embrace the toolkit that best positions us to do so’ (W1, P11). Linked to questions of effectiveness, procedural criteria relate to the definition of goals, aspects of evaluation or the professional use of communication instruments, and there is a ‘need to be wary of inadvertently conflating “measurable” with “quantitative.” This seems to happen a lot unless explicitly considered’ (W2, P17).

**Table 2.** Meta-criteria for assessing science communication quality.

Meta-criteria	Description	Most important criteria
Content	What is communicated?	Relevance, accuracy, completeness, objectivity truthfulness
Presentation	How is it communicated?	Accessible language and style, engaging communication
Technical	How does the infrastructure interact with the communication?	Opportunities for dialogue and feedback, technical accessibility
Context	What is the context of communication?	Clear purpose/motivation, expertise of sources, transparency, reliability of evidence
Process	What precedes/follows the communication?	Definition of goals, standards evaluation

### Context dependency of quality assessment

Whereas the framework of meta-criteria gives an initial suggestion of ways to assess science communication quality online, many experts stressed the context dependency of quality assessment (Lacy and Rosenstiel, 2015): ‘I think “quality” is only meaningful when related to aim/goal/aspiration. What makes for a quality post on I Fucking Love Science won’t cleanly, clearly or necessarily apply to what makes for quality content on the New York Times’ (W1, P12). Experts argue that any quality assessment requires knowledge of the actual context of the respective science communication setting. To explore this perspective, we asked experts for the validity and applicability of quality criteria for different settings of science communication online. We suggested six settings that differed in terms of communicator, channel, and purpose and asked experts to compare two of them regarding quality criteria and to explain which criteria would be relevant and which would not apply. We proposed the following settings:

(a) A news section on a university website presenting the latest research from their organization.
(b) The Twitter thread of a scholar commenting on policy issues by referring to the latest evidence.
(c) A governmental campaign on different social media referring to public health issues.
(d) The blog of environmental activists citing scientific studies to strengthen their argument.
(e) An influencer’s post on Instagram presenting spectacular scientific experiments.
(f) A podcast provided by the science section of a leading daily newspaper.

Some experts found responding to this question challenging and skipped it. Reported responses led to a variety of comparisons of settings and associated criteria. For instance, the comparison of settings A (university website) and B (Twitter thread) was strongly related to the communicators’ capability to ‘achieve their objectives/goals in ethical ways’ (W2, P13). An expert comparing settings A and C argued that,

in A (university website) content and context criteria would be most important, in particular, transparency about the funding of the research; as well as presenting it truthfully and without distortion (no hype or overselling). [. . .] In C (government campaign), presentation criteria would be paramount (making it engaging, clear, brief, and simple and catering for diverse audiences), but you cannot exclude the other criteria. (W2, P6)

Different settings ‘obviously speak to very different criteria [but all] however should be judged on how successfully they connect with their audiences’ (W2, P2).

Assessing quality for the different settings was regarded as a ‘matter of relative importance of different criteria in different settings, [. . .] [rather] than a case of some not applying. They all apply, to a greater or lesser extent’ (W2, P6). This statement encapsulates a somewhat contradictory result: on the one hand, participants found it very challenging to identify quality requirements for different situational settings, but on the other hand, they largely rejected the idea of generalizable criteria. Many experts who had first claimed the context dependency of quality assessments now stressed the difficulty of distinguishing different settings although for different reasons. Whereas one participant stated that the assessment of quality was first and foremost a question of the purpose of the communication, other respondents emphasized the ‘intense competition for attention’ (W2, P12) as a core factor arguing that any ‘quality assessment is in the eye of the audience’ (W2, P8). Another respondent stated that the multiple possible modes of interaction of communicators and platforms would make quality assessments difficult. Moreover, the level of ‘controversy or urgency’ (W2, P15) of the topic being communicated was introduced as an intervening factor, especially against the backdrop of COVID-19.

Another surprising outcome was that experts chose only those situational settings with which they are likely to be most familiar: the university website (*N* = 6), the scholars’ thread on Twitter (*N* = 7) and the podcast of the newspaper (*N* = 5). Although chosen less often, the government campaign was considered (*N* = 4). In contrast, the situational settings D (*N* = 1) and E (*N* = 1), the blog of environmental activists and the Instagram post, were hardly discussed. As these examples differ most from journalistic and academic approaches to science communication, it would have been particularly interesting to gather scholars’ thoughts on which criteria could be applied to them. This raises the question of whether experts’ perspectives keep pace with developments in science communication in the digital media world.

### Promoting quality in science communication online in the future

**Figure 1.**
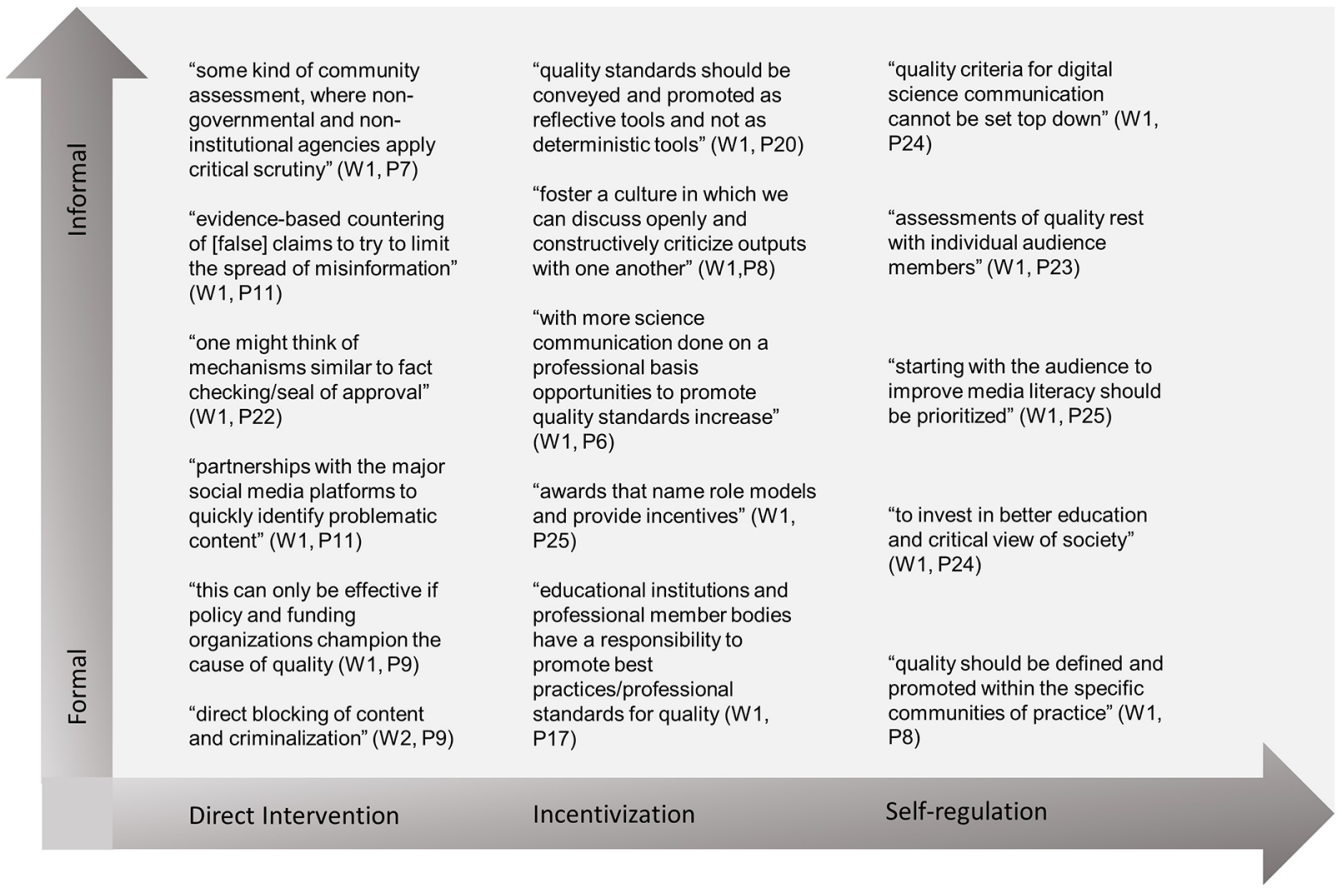
Experts’ positions on ensuring quality indicators.

We asked how experts would convey, promote or even secure the quality criteria that they considered most important. Figure 1 outlines different positions in the quality discourse. The map indicates that experts hold diverse and even contradictory positions on how to promote, convey or secure quality indicators. Different arguments can be located on a continuum from direct intervention (e.g. ‘fact checking’, collaboration with/regulation of platforms (W1, P22)) to self-regulation (e.g. ‘quality standards should be conveyed and promoted as reflective tools and not as deterministic tools’ (W1, P20)), with incentivization (‘the best we can hope for is to foster a culture in which we can discuss openly and constructively criticize outputs with one another’ (W1, P8)) in between the poles. Another distinction can be made between formal and informal approaches.

Experts were also asked which of these approaches were used. Many participants responded that all formats were used, ‘albeit to different degrees’ (W2, P4), and listed examples for the different approaches. Many experts emphasized differences in national and political contexts, thus stressing the societal and political embeddedness of science communication: ‘In authoritative countries, we have strict regulation and suppression of digital (science) communication. In western countries with liberal democracies, incentivisation and self-regulation dominate’, with few opportunities for ‘regulation appearing between governments and social media companies’ (W2, P14). In these political contexts, experts state that approaches to incentivization and self-regulation are particularly visible. Experts also argue that the increasing science communication training of scientists and the growing demand for outreach and public engagement activities ‘as part of research funding and assessment contributes to best standards’ (W2, P12).

### Roles and opportunities

**Figure 2.**
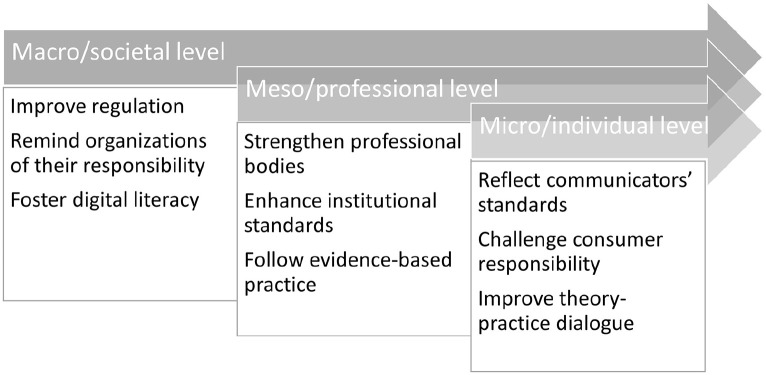
Experts perspectives on responsibilities for science communication quality.

Finally, we asked experts which approaches had the potential to facilitate the implementation of quality standards. Overall, the responses refer to different approaches, which, as one participant states, are not mutually exclusive. Instead, ‘a combination of various interventions working at the same time’ (W2, P12) is needed. Based on the results, there are three perspectives which are considered especially relevant and which can be mapped to a macro-, meso- and micro-perspective (Figure 2).

On the macro level, experts locate the responsibility to promote science communication quality at a societal level ‘including government, society and social media companies’ (W2, P2), with the assumption that ‘the key thing is for social media companies to understand their role in society and the ethics surrounding their responsibility’ (W2, P2), though blocking content was also seen to present a threat. Instead, content moderation should develop in a ‘democratic system’ (W2, P15). Respondents stress the need to start and engage in social discourses, for example, regarding ethical requirements and regulation of social media communication. Furthermore, education should foster and improve digital literacy. Approaching these tasks of ‘building competency and literacy’ (W2, P15), however, should be undertaken with the communities themselves, including schools and broader public education. In addition, ‘to promote and encourage scholarly discussion and reflection’ (W2, P6), by means of research like ours, is considered as an important starting point.

On a meso level, experts refer to professional bodies, science communication societies and associations, and scientific institutions and their role in quality assurance. Their contribution is seen as providing ‘background knowledge’ (W2, P10). By integrating questions of quality in their ‘internal debates’ (W2, P15) and by the ‘establishment of standards and education’ (W2, P13), they could contribute further to the promotion of science communication quality. Whereas the professional development of science communication has been discussed controversially (Bauer, 2013), one expert argues that linking professional communication with quality would be valuable. A stronger focus on quality, ‘may help to overcome a recent trend in seeing some “stigma” attached to science communication as being a mere “PR” exercise for scientific institutions [. . .]’ (W2, P6). To address this, ‘in some countries, the professional bodies overlap (e.g, SCOM with sci journalism or with PR), and in [this] overlap, there are opportunities to foster much better learning for members of such organisations’ (W2, P16). This exchange with other communities (science journalism, science public relations) is regarded as especially valuable. Professional development is also linked to an evidence-informed practice of science communication which,

should be based on social scientific evidence [so] that messages will likely be effective or have been proven to be effective in other contexts. We need to stop the trial-and-error nonsense by social and bench scientists who think they ‘know what works’ or that they ‘are good at this’. (W2, P15)

On a micro level, experts address the responsibilities of science communication professionals and scholars, scientists, lay actors who communicate science and users of digital science communication content. Science journalists and PR experts, for example, are supposed to align with the standards and demands defined by their professional communities to ‘justify their choices’ (W2, P13). Science communication professionals are also considered as a kind of facilitator in collaboration with scientists, relieving them of responsibility to a degree as scientists should not ‘be expected to do everything on their own just as we don’t expect politicians or executives to manage all their own communication’ (W2, P14). Instead, professional assistance would help maintain quality standards, ‘especially in cases where there’s a need for robust planning, implementation, and evaluation’ (W2, P14). A few respondents refer to influencers or activists who contribute to the public perception of science and therefore should also conform to quality standards, though they do not clarify what this would mean. There was also a recognition that trusted sources depend on topic and context and that ‘anyone communicating in their professional role should have to justify their choices just like researchers and instructors’ (W2, P13). Similarly, ‘consumers of science communication cannot disregard their own responsibility’ (W2, P7). Finally, science communication scholars are expected to contribute to science communication quality, by ‘advance[ing] conceptual understanding and theory’ (W2, P10) and thereby contribute to the development of approaches for explanation. Furthermore, scholars should ‘provide empirical evidence’ (W2, P10) to observe and reflect upon the development of science communication practice. Respondents emphasized that collaboration with practitioners should be strengthened, for example, to ‘provide material for reflection’ (W2, P8), to ‘collaborate in research projects’ (W2, P11) and to use knowledge to ‘solve specific practical problems’ (W2, P13).

## 5. Conclusion and Outlook

The COVID-19 crisis has highlighted the importance of science communication and raises questions about how we assess quality. The starting point of this study was the changing digital science communication landscape, which is facilitating access to scientific information in new ways and enabling a more diverse range of participants to communicate science. Science communication can no longer be reduced to science journalism with its quality standards, but today encompasses all public communication about science. This diverse landscape not only holds many opportunities for the interaction of science and society but also poses threats and challenges, such as misinformation and interest-driven communication. Here, we presented a quality framework for digital science communication and identified strategies to promote quality indicators. The framework could provide scientists and researchers, professional science communicators, decision makers and laypersons with assistance and orientation in the evaluation of science communication. The framework points to five meta-criteria through which communicators and readers can begin to make judgements about the quality of the material they produce and consume. From a practitioner’s perspective, these five meta-criteria provide a useful framework through which to assess their communication output. For consumers, the framework offers criteria through which digital science communication could be evaluated. For scholars, further work could explore how these meta-criteria are adapted to fit particular digital contexts and their usefulness for publics and practitioners. The research also highlights the need for educational approaches to help consumers assess quality.

The study produced some surprising results. We were astonished that so few experts considered emerging digital contexts, instead focusing on well-studied science communication environments, such as journalism, PR and scientists’ public engagement. Respondents missed the opportunity to consider quality criteria that would apply, for example, to the communication of influencers or NGOs on social media. This focus on forms of science communication that have already received considerable scholarly attention is striking and hints at a gap in reflection around the ‘new’ digital settings of science communication, ones which arguably pose the greatest challenges for ensuring quality and thus deserve closer attention, analysis and reflection. This was even more surprising, as scholars are often involved in policy consultations to tackle quality concerns in science communication.

There were significant differences in the participants’ attitudes to the fundamental question of whether quality criteria can be determined at all. Some participants insisted throughout the study that quality criteria are so context-dependent, that identifying a generalized set of criteria is nearly impossible. Addressing this, the quality framework presented here is flexible, allowing for adaptation to recognize differing contexts across platforms and potentially countries. When considering strategies for the implementation of these criteria, experts agree not only on the need for education but also for reflection and raising awareness within the science communication community. Strengthening the collaboration between scientists and practitioners to evaluate the quality discourse is considered an important approach. Other approaches, such as fact checking or content flagging approaches, were valued by some, but raise questions around freedom of speech and whether such services might inhibit wider societal discussion of scientific and technological issues. In addressing these points around implementation, we suggest considering what approaches might be addressed at macro (e.g. education), meso (e.g. professional development) and micro (e.g. individual communicator) levels.

The study focused on the assessment of science communication scholars. We took this approach to ensure a certain degree of comparability within the panel. Nevertheless, there was great heterogeneity among the perspectives on quality criteria and considerable controversy with respect to some approaches suggested for implementation. Future research should focus on these contradictions and seek input from the practitioner community on the concerns they have with regard to quality or implementation of quality indicators. As our quality ratings are based on experts’ assumptions, we cannot say anything about users’ ratings, another area for exploration. Systematic follow-up research on reception and impact would be important here.

We see our study as a stepping stone in the discussion about quality indicators for science communication. A conversation about these issues should not only take place within the science communication community but also in science, politics and society at large.

## Supplemental Material

sj-docx-1-pus-10.1177_09636625221148054 – Supplemental material for Exploring ‘quality’ in science communication online: Expert thoughts on how to assess and promote science communication quality in digital media contexts Click here for additional data file. Supplemental material, sj-docx-1-pus-10.1177_09636625221148054 for Exploring ‘quality’ in science communication online: Expert thoughts on how to assess and promote science communication quality in digital media contexts by Birte Fähnrich, Emma Weitkamp and J. Frank Kupper in Public Understanding of Science

## Notes

1. Additional data from the same study were used for a paper (Fähnrich, 2021) discussing perspectives on how to define science communication against the backdrop of the digital transformation.

2. To be consistent and due to the different national settings of respondents, the Wave 2 questionnaire did not refer explicitly to quality issues in the context of COVID-19. However, it should be acknowledged that experts’ assessments were probably influenced by the developments as concerns about the quality of science communication received greater public and political attention during the pandemic. With this in mind, it is possible that the results from Wave 2 have been affected by the pandemic.

3. Quotations indicate the waves from which statements derive (Wave 1 or 2), and reference the experts who are randomly numbered for Waves 1 and 2 (e.g. W1, P3).
