# Supplementary material for: Exploring ‘quality’ in science communication online: Expert thoughts on how to assess and promote science communication quality in digital media contexts
Source: Public Underst Sci. 2023 Jan 31;32(5):605–21. doi: 10.1177/09636625221148054 (PMC10336610; doi:10.1177/09636625221148054)
Supplement: sj-docx-1-pus-10.1177_09636625221148054 – Supplemental material for Exploring ‘quality’ in science communication online: Expert thoughts on how to assess and promote science communication quality in digital media contexts [file sj-docx-1-pus-10.1177_09636625221148054.docx]

Exploring ‘quality’ in science communication online

Expert thoughts on how to assess and promote science communication quality in digital media contexts

Supplemental Material

Birte Fähnrich^1^, Emma Weitkamp^2^, J. Frank Kupper^3^

^1^ Department of Political and Social Sciences, Freie Universität Berlin (Germany)

^2^ Science Communication Unit, University of the West of England, Bristol (Great Britain)

^3^ Athena Institute, Vrije Universiteit Amsterdam, Amsterdam (Netherlands)

**Questionnaire – Wave 1**

Thanks for your participation in our research. This Delphi study seeks to investigate the meaning of quality in the context of science communication within the digital media environment. The Delphi study is split into two waves. This email solicits your input for the first wave, which explores the overall conceptualisation of science communication in the context of digital transformation. The questionnaire, therefore, entails three short sections with open questions, which we anticipate will take approximately 30 to 45 minutes to complete.

Based on the different disciplinary backgrounds and academic interests of the panellists, we assume that responses to the questionnaire will result in a variety of perspectives. We will analyse your responses using a summarising and structuring approach and use the results as a basis for the second and final wave of the Delphi, which will be carried out in March of 2020.

We have once again attached the information sheet for the Delphi study, which also includes information on funding, anonymity, data security and ethical concerns.

Should you have queries, please do not hesitate to contact us.

1. ***A. Conceptualising science communication in the context of digitalisation***

There is a global consensus that the spread of digital media has profoundly changed public communication. This is also true in relation to science communication, which is evident in the immense pluralisation of science-related content, the fragmentation of audiences and the increasing differentiation of professional and lay communicators, to name but a few developments. In the digital media environment, science communication has developed from a basic journalistic domain into a patchwork of highly heterogenous and complex phenomena of public communication. This patchwork encompasses journalistic formats as well as an array of science-related content on websites, blogs, wikis or platforms such as YouTube, Instagram, etc.

Against this backdrop, we would like to start this Delphi study with a conceptualisation of science communication in the digital media environment. We would thus like to ask you to share your perspective on what science communication means to you. There are no right or wrong answers, as our purpose is to look for a definition as a starting point:

**Q1: How would you define science communication in the digital media environment?**

***B. Conceptualising quality in the context of digital science communication***

Digitalization has enabled considerable diversification in the area of science communication, e.g. in terms of communicators, formats, issues, etc. We observe that science communication originating from different types of communicators (e.g. professional communicators, enthusiastic lay people) and employing very different platforms (blogs, YouTube channels, Instagram) also vary in terms of quality. Considering this context, please provide your views on the following questions:

**Q2: What are the most important quality criteria for evaluating science communication online?**

**Q3: Where could quality criteria for science communication be drawn from (e.g. journalism, science, public relations, other)?**

***C. Quality assurance and promotion of standards***

Thinking about the quality of science communication in an increasingly diverse digital media environment raises questions about how to also ensure quality in science communication. Please share your views on quality assurance in the digital science communication environment.

**Q4: Should there be a quality assessment of science communication outputs in the digital media environment? If so, what might such an assessment look like, and who should be in charge?**

**Q5: How could quality standards for science communication in the digital media environment be conveyed and promoted?**

***D. Sociodemographic information***

**1) Please name the country in which you are currently employed?**

**2) Please indicate your disciplinary background.**

Communication science

STS

Media Studies

Psychology

Political Science

Other, please indicate

**3) What is the level of your current position?**

Pre-doc/junior researcher

Assistant professor/postdoc/senior researcher

Associate/full professor

Thank you for your support!

**Questionnaire wave 2**

Dear participant,

Once again thank you for taking part in our DELPHI study on the “Quality of Science Communication in Digital Contexts!

The first wave of our study that was answered by 26 international and interdisciplinary scholars aimed at collecting a broad range of different perspectives and assessments on the issue in focus. To this end, we asked you how you would conceptualize/define science communication online, which were the most important quality criteria and how these could be promoted, conveyed or even regulated. We used this very open and explorative approach because we assume that we cannot simply adopt to existing criteria and apply them to new science communication phenomena, but need a fundamental reassessment of science communication quality in a digital context.

In the analysis of your responses, we summarized, ordered and interpreted results by using the situational analysis approach. Situational analysis is a method developed by Adele Clarke (2003, cf. Clarke et al. 2018) that allows analysis of elements and the complex social constellations they are embedded within by using different forms of visual maps. These visualisations are not only applied as analytical tools, but are also used in the results alongside quotes. Quotes are demarcated in the usual way.

With this second wave, we ask you to comment on and add to the preliminary results. We will thus present our interpretations of your thoughts and the breadth of your perspectives to order, focus and to back up the findings.

Again, the questionnaire will comprise three sections that build upon each other. Based on your previous responses, it will start with a further clarification of the notion of “science communication in digital contexts“, it then moves on to refine the quality criteria that emerged from your responses as applied in different situational settings and eventually asks you to reflect upon approaches to promote these criteria in the online science communication landscape, as identified through the initial questionnaire responses

Thanks for taking the time to respond once again!

1. *I. Conceptualizing Science Communication in Digital Contexts*

In the first wave, we asked you to define science communication against the backdrop of the digital transformation. We did so because existing definitions vary considerably and were not developed with digital contexts in mind. We received quite a broad range of responses. Whereas some of you indicated that the digital context would not necessarily change the overall concept of science communication, others argued that the tremendous changes afforded through digital tools would require “a new - and presumably ongoing - conceptual understanding“ (P3). We would like to interrogate this perspective further.

Different participants emphasised the diversification of science communicators, channels, forms and contexts in the digital environment concluding that we should not “define ‘science communication` too narrowly“ and that, against the backdrop of the digital media environment, “the definition of science communication comes down to the *content* being communicated (or not)“ (P24). Drawing on your responses, we would propose the following definition:

**Science communication in digital contexts encompasses all forms of communication about science-related topics via digital media.**

This definition is descriptive and broad and thus helps to cover the entire digital science communication landscape reaching from professional science journalism or university communication on websites, blogs and public engagement by scholars via twitter to diverse forms of science-related communication whether by science enthusiasts or deniers on YouTube, reddit or other platforms. However, we acknowledge that such a broad definition is hardly useful as an actual working definition. Instead, we need to draw at least “fuzzy boundaries“ (P19) to distinguish different situational settings of science communication in digital contexts.

Such a situational setting could encompass, for instance, political actors that aim at changing people’s behaviour with regard to health issues and thus use a public information campaign that is run and shared on diverse online channels. A completely different setting would entail a company which applies latest findings from nutritional science in their PR communication on different social media to improve its overall reputation. Another situational setting again could refer to a scientist involved in citizen science who uses online tools to foster a mutual learning experience with a group of lay participants.

| As there are numerous of these situational settings, we think that it is useful to systemize the field on a more general level. Therefore, we aimed at developing a matrix to distinguish different settings of digital science communication. To this end, we analyzed your responses from wave 1 and inductively built categories. On this basis, situational setting of digital science communication are shaped by different combinations of the following (and potentially further) categories: **Actors involved** (e.g. scientists, journalists, politicians, engineers, industry, citizens, PR offices, NGOs) |
| --- |
| **Roles taken by these actors** (e.g. knowledgeables, lay, professional, non-professional, authority, communicators, multipliers, recipients) |
| **Intentions of the actors** (e.g. information, increase science literacy, learning, attitude & behavioural change, entertainment, fun) |
| **Level of formality of communication** (e.g. organized actions, spontaneous communication) |
| **Content** (e.g. aspect of science such as knowledge, findings, practices & processes, methods, scientists, scientific expertise, science policy) |
| **Platforms used** (e.g. websites, blogs, social media) |
| **Level of convergence** (e.g. limited to few platforms, shared on many platforms) |
| **Modes of communication** (e.g. informational, popular, dialogic, interactive, controversial, discursive, referring to opinions, ideas and/or facts) |
| **Types of presentation** (e.g. text, audio, video) |
| **Levels of publicity** (e.g. closed groups, clearly defined publics, diverse publics) |
| **Types of effects** (e.g. with regard to changes of knowledge, attitude behaviour, measurable/not measurable, shortterm/longterm) |
